# Mucoadhesive Polyvinyl Alcohol Film Containing *Fridericia chica* Extract for Mucositis Treatment: Pharmacological Evaluation and Active Compounds Release

**DOI:** 10.1002/cbdv.202502095

**Published:** 2025-10-25

**Authors:** Ilza Maria de Oliveira Sousa, Ailane Souza de Freitas, Fabrício de Faveri Favero, Lucia Elaine O. Braga, Mariana Cecchetto Figueiredo, Diana Pinto, Maria Beatriz P. P. Oliveira, Simone Nataly Busato de Feiria, Nubia de Cássia Almeida Queiroz, Ana Lucia T. G. Ruiz, José Francisco Hofling, João Ernesto de Carvalho, Mary Ann Foglio

**Affiliations:** ^1^ Graduate Program in Medical Sciences, School of Medical Science (FCM) Universidade Estadual de Campinas (UNICAMP) Campinas Brazil; ^2^ School of Pharmaceutical Sciences (FCF) Universidade Estadual de Campinas (UNICAMP) Campinas Brazil; ^3^ Department of Physiological Science—Piracicaba Dental School (FOP) Universidade Estadual de Campinas (UNICAMP) Piracicaba Brazil; ^4^ LAQV/REQUIMTE, Faculty of Pharmacy University of Porto Porto Portugal

**Keywords:** 3‐deoxyanthocyanidins, *Arrabidaea chica* Verlot, healing, mucositis, permeation

## Abstract

Oral mucositis is a frequent and painful complication of cancer therapy with limited effective options. This study evaluated a mucoadhesive polyvinyl alcohol (PVA) film containing 2.5% *Fridericia chica* extract as a novel strategy. The formulation was characterized for antioxidant activity, phenolic and flavonoid content, and reactive species scavenging. Nanostructure size and morphology were assessed by DLS and TEM. Biological assays included antimicrobial activity, ex vivo release (Franz cells), and cytotoxicity in HGF‐1 and HaCaT cells. The film showed antioxidant and antimicrobial activity against *Candida* spp. and bacterial strains, and showed no cytotoxicity up to 62.5 µg/mL. Particles were nanometric (245.5 nm), monodisperse, with a zeta potential of −11.13 mV. 3‐Deoxyanthocyanidins and luteolin permeated the porcine esophageal membrane, indicating that PVA did not hinder compound release. The *F. chica* + PVA film may improve mucosal adhesion and sustain delivery, representing a promising alternative for mucositis treatment.

## Graphical abstract

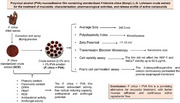

## 1 Introduction

Yearly, over 660 000 people worldwide receive head and neck cancer diagnosis, which is ranked as the seventh most common type, with 325 000 deaths [1, 2]. The complication rates associated with these types of tumors are high. Data from a systematic review showed that the incidence of oral mucositis in patients with head and neck cancer ranges from 59.4% to 100% of cases, with varying degrees of severity [3]. Oral mucositis is a painful and debilitating condition characterized by erythematous inflammatory process in oral cavity causing pain and hindering deglutition and speech. The risk of malnutrition and infections increase considerably in patients with this condition, hindering treatment with reduced quality of life [3, 4, 5].

Mucositis treatment has focused on pain management, with the administration of analgesics and low‐intensity laser therapy (LLT) [4, 6]. Natural products are a source of bioactive compounds with unique structures that can provide new treatments. Approximately 60% of new drugs approved between January 1981 and January 2019, directly or indirectly derive from natural products [7]. *Fridericia chica* (Bonpl.) L.G. Lohmann (syn. *Arrabidaea chica* Verlot) is popularly known by “crajiru” or “pariri” in Brazil. This species is widely distributed, occurring in all the country's phytogeographic domains [8].

Brazilian Indigenous communities traditionally used *F. chica* leaf extract as a natural dye for body painting and as protection against sunlight and insects [9]. In folk medicine, leaf infusions are employed to treat colic, diarrhea, and anemia [10]. Scientific evidence further supports the plant's therapeutic potential for wound healing, gastrointestinal ulcers, and colic [11, 12, 13]. Our group has demonstrated wound‐healing effects through preclinical and clinical studies, including in vitro fibroblast and keratinocyte assays and in vivo models showing increased collagen deposition and improved healing of mucosal and skin ulcers [14, 15, 16]. A Phase II/III clinical trial is currently in progress.

In 2009, the Brazilian Ministry of Health published the List of Medicinal Plants of Interest to the National Health System (RENISUS) encouraging research with medicinal plants for access and promotion of health benefits, with *F. chica* among this list [17]. The leaf extract exhibits an overall red color due to chromatic properties of 3‐deoxyanthocyanidins: 6,7,3′,4′‐tetrahydroxy‐5‐methoxy‐flavylium, 6,7,4′‐trihydroxy‐5‐methoxy‐flavylium, and 6,7‐dihydroxy‐5,4′‐dimethoxy‐flavylium (carajurin), the latter being the plant's main chemical marker [18]. Also, luteolin, apigenin, and scutellarein contribute to the extract's healing, antimicrobial, anti‐inflammatory, and anticarcinogenic properties [19, 20].

Studies with the standardized extract dried by spray drying into a semi‐solid base, demonstrated in vivo healing properties on skin ulcers [21]. The extract also presented antimicrobial activity against *Enterococcus faecalis*, *Helicobacter pylori*, and *Candida* spp. [22, 23, 24]. The healing potential of *F. chica* encouraged a randomized clinical study, which allowed volunteers undergoing radiotherapy treatment for head and neck cancer to treat mucositis with natrosol gel containing 2.5% standardized extract, administered topically at the lesion site [4].

Among challenges in the development of topical formulations for mucosal treatment is adhesion duration [25, 26]. For oral cavity treatments, bioadhesive preparations have the advantage of adhering to the mucosa, thereby protecting the affected area. Among the different materials employed in biofilms is polyvinyl alcohol (PVA), an efficient mucoadhesive polymer, forming flexible, elastic, and permeable films with high compatibility with pigments and wetting agents [27]. To enable the application of *F. chica* extract in clinical study some formulations were evaluated [4, 28]. This study describes ex vitro extract release profile studies and characterization data of the mucoadhesive PVA film (PVA) containing 2.5% *F. chica* standardized crude extract (SCE).

## 2 Results and Discussion

### 2.1 Chemical Characterization

The generation of oxidative stress results from an imbalance between oxidizing and antioxidant molecules in the body. Reactive species (ROS) are crucial for cellular homeostasis; however, when produced in excess, they contribute to the development of diseases such as cancer, diabetes, hypertension, and inflammatory conditions like mucositis [10]. Oral mucositis is triggered initially after chemotherapy treatment with development of erythema, accompanied by the presence of free radicals, immune cells, and inflammatory cytokines. Following the primary immune response, signal amplification occurs, compromising the integrity of the mucosa and leading to ulceration. In the final phase, healing takes place, with mucosal restoration and angiogenesis. Antioxidant compounds play a key role in neutralizing the free radicals formed during chemotherapy, potentially preventing the formation of mucositis and supporting the healing process [29, 30].

The *F. chica* SCE was capable in attenuating oxidative stress as result of total phenolics and flavonoids amount (Table 1). The *F. chica* + PVA film exhibited higher levels of phenolic compounds (215.9 ± 3.8 mg GAE/g; *p* < 0.001) and flavonoids (97.9 ± 1.8 mg CAE/g; *p* < 0.001) than the *F. chica* SCE (Table 1), demonstrating that PVA, when incorporated into the crude extract, efficiently protected these compounds from exogenous factors such as light, temperature, humidity, and oxygen [31], consequently enhancing the extract's antioxidant activity. The extraction method influenced the phenolic content, with a direct relationship among phenolic and flavonoid compounds and the method used. Jorge et al. [14], using methanol to prepare the *F. chica* extract, reported 67.69 mg GAE/g, lower than in the present study. Port's et al. [31], who prepared an aqueous infusion of *F. chica* leaves, found 43.20 mg GAE/g and 7.78 mg CAE/g, also lower than our results. Similarly, Sousa et al. [32] used acidified 70% ethanol and spray drying to obtain 143 mg GAE/g and 48.08 mg CAE/g, in agreement with our study.

**TABLE 1:** Evaluation of total phenolic content, total flavonoid content, and antioxidant activity of *Fridericia chica* SCE and *F. chica* + PVA film.

Assay	*F. chica* SCE	*F. chica* + PVA film	Positive control (Trolox)
Total phenolic content (mg GAE/g sample)	139.6 ± 3.7	215.9 ± 3.8^***^	n.a.
Total flavonoid content (mg CAE/g sample)	56.4 ± 1.1	97.9 ± 1.8^***^	n.a.
DPPH (IC_50_ µg/mL)	449.8 ± 2.7	393.4 ± 21.4^**^	62.7 ± 0.9
FRAP (µmol Fe^2+^/g sample)	1.48 ± 0.01	1.77 ± 0.02^***^	n.a.

*Note*: Results expressed as mean ± standard error of the mean (SEM) of three independent assays, in three technical replicate per concentration. Samples: *F. chica* + PVA film = PVA film loaded with 2.5% of *F. chica* SCE; *F. chica* SCE = standardized crude extract of *F. chica* leaves. Statistical analysis: paired *t* test (*F. chica* SCE vs. *F. chica* + PVA).

Abbreviations: CAE, catechin equivalent; GAE, gallic acid equivalent; IC_50_, sample concentration required to reduce 50% of the DPPH concentration; n.a., not applicable.

** *p* < 0.01.

*** *p* < 0.001.

### 2.2 Antioxidant Activity

In general, higher phenolic compound content correlates with greater antioxidant activity. This can be observed in the FRAP and DPPH results between *F. chica* + PVA film (1.77 ± 0.02 µmol Fe^2+^/g, *p* < 0.001 and IC_50_ = 393.4 ± 21.4, *p* < 0.01), which showed better values compared to *F. chica* SCE (1.48 ± 0.01 µmol Fe^2+^/g extract and IC_50_ = 449.8 ± 2.7) (Table 1). Previously, our research group obtained IC_50_ values of 466.99 and 445.6 µg/mL, FRAP of 1.45 and 1.72 µmol of FSE/g for the extracts dried in a spray dryer, with and without a nitrogen atmosphere, respectively [32]. These results indicate moderate antioxidant potential.

The evaluation of antioxidant activity using the DPPH molecule is widely reported due to the compound's good stability in the absence of light, along with the method's simplicity and applicability. However, this assessment should not rely on a single methodology, and additional assays, such as the FRAP method, are necessary for a more comprehensive characterization of an antioxidant compound. DPPH assay protocols are often modified to adapt the dissolution solvents, initial concentrations, reaction time, and absorbance readings, making comparisons between results challenging [33, 34]. In this study, the method was limited by the lower solubility of DPPH in the solvent used (70% ethanol), as the radical dissolves better in organic solvents like absolute ethanol and methanol. In contrast, the crude extract of *F. chica* has better solubility in 70% ethanol.

Both *F. chica* + PVA film and *F. chica* SCE demonstrated considerable free radical scavenging potential (Table 2), particularly for hypochlorous acid. This effect has relationship with the high content of polyphenols and flavonoids, especially 3‐deoxyanthocyanidins [32]. There was no statistical difference in the hydrogen peroxide reduction capacity, with IC_50_ values of 439.2 ± 16.7 for *F. chica* SCE and 406.4 ± 14.1 for *F. chica* + PVA film. In contrast, the crude extract showed better results for superoxide anion reduction (38.3 ± 1.27, *p* < 0.05) and hypochlorous acid reduction (3.72 ± 0.03, *p* < 0.01).Although H_2_O_2_ is one of the least ROS, this radical can generate other species with more powerful and toxic effects, such as the hydroxyl radical (HO^−^) and hypochlorous acid (HClO) [32, 35].

**TABLE 2:** Scavenging capacity of superoxide anion radical (O_2_^−^), hydrogen peroxide (H_2_O_2_), and hypochlorous acid (HClO) of *Fridericia chica* SCE and *F. chica* + PVA film.

	IC_50_ (µg/mL)			
ROS	*F. chica* SCE	*F. chica* + PVA film	Positive controls	
			Gallic acid	Quercetin
H_2_O_2_	439.2 ± 16.7^a^	406.4 ± 14.1^a^	202.9 ± 9.2^b^	—
O_2_^−^	38.3 ± 1.27^a^	107.4± 9.9^b^	13.8 ± 0.3^a^	—
HClO	3.72 ± 0.03^a^	6.23 ± 0.17^b^	3.5 ± 0.16^a^	0.23 ± 0.006^c^

*Note*: Results expressed as mean ± standard error of the mean (SEM) of three independent assays, in technical triplicate per concentration. Samples: *F. chica* + PVA film = PVA film loaded with 2.5% of *F. chica* SCE; *F. chica* SCE = standardized crude extract of *F. chica* leaves. Statistical analysis: ANOVA and Tukey's test. Means followed by the same letter do not differ significantly by the Tukey's test at *p* < 0.05.

Abbreviation: IC_50_, sample concentration required to reduce 50% of the reactive oxygen species (ROS) concentration.

a: ***p < 0,001; b: **p < 0,01; c: *p < 0,05.

The effects of *F. chica* crude extract are not solely attributed to its antioxidant potential. The extract also promotes increased fibroblast proliferation, collagen synthesis induction, and a reduction in pro‐inflammatory cytokines, such as TNF‐α, IL‐6, IL‐4, and IL‐10, which are secreted during mucositis formation, thereby preventing the inflammatory effects of these mediators in ulcer generation [13, 14]. These properties, combined with the plant's antioxidant potential, work synergistically to preserve mucosal integrity, reducing severity, and mucositis span [29, 30]. Timely treatment is crucial for oncology patients, as they experience pain and discomfort during both the acute and chronic stages of oral mucositis.

In general, as consequence to mucositis, there is a necessity to interrupt anticancer treatment, as well as to provide parenteral nutrition and support with other medications, such as antimicrobials and analgesics. This condition often results in prolonged hospital stays for the patient, leading to higher economic burdens on the healthcare system [36, 37]. A systematic review conducted in 2019, which included studies on mucositis from the Multinational Association of Supportive Care in Cancer/International Society of Oral Oncology (MASCC/ISOO), recommended combined oral hygiene protocols, photobiomodulation, and, in specific cases, cryotherapy. To date, low‐level laser therapy (LLT) remains the most recommended intervention for prevention and treatment, since the procedure alleviates pain and edema symptoms. The advantage of laser treatment lies in the absence of reported adverse effects, along with being noninvasive and painless [37].

However, the patient may experience difficulty opening their mouth due to the extent of the wound, as well as symptoms of nausea and vomiting post‐chemotherapy, which can reduce the efficiency of the laser treatment [37]. The use of laser not only involves purchasing the low‐power device but also consumables, requiring significant financial investment, biosafety materials, and qualified professional for handling and application, which is the most financially impactful requirement [36]. The total cost per patient depends on the number of sessions and the prevalence of each type of cancer. From the perspective of the Brazilian Health System, hematologic neoplasms affect approximately 10 857 adult individuals annually. Given the high incidence of mucositis in these patients, the cost of administering the therapeutic protocol (10 sessions) would be BRL 269 503.13 for the year 2022. Considering the incidence of both head and neck and hematological cancers, in a best‐case scenario where patients undergo only five sessions, the annual financial impact on the healthcare system would be BRL 2 225 630.31 [36].

However, the most significant impact is not economic. The need to travel to the hospital and seek professional help for treatment and prevention complicates the proper management of mucositis. Formulations with *F. chica* SCE could help address this issue, as patients can apply for the product at home after receiving administration instructions. In a Phase II/III clinical study, participants with onco‐hematological diseases undergoing bone marrow transplants were randomized to treat mucositis with either 2.5% *F. chica* mucoadhesive gel or low‐level laser therapy. Preliminary results showed that the group treated with *F. chica* healed, in average, in 5.5 ± 0.9 days, while the laser‐treated group took, approximately, 11.4 ± 3.6 days. Using a visual analogy scale, patients reported average pain levels, with the *F. chica* group scoring 0.1 ± 0.3 points and the laser group scoring 1.6 ± 2.1 points at the end of treatment. These findings suggest that *F. chica* is effective, offers a shorter healing time, and is more convenient and economical [38]. Larger patient groups are currently being studied by our research team, with results to be published elsewhere.

### 2.3 *F. chica* + PVA Film Characterization

**FIGURE 1:**
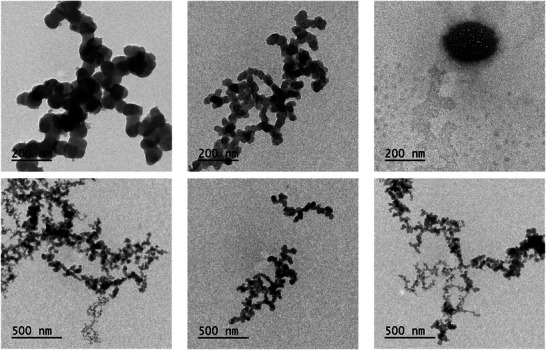
Representative microphotograph of *Fridericia chica* + PVA film obtained by transmission electron microscopy (TEM).

Dynamic light scattering (DLS) and the morphology of the particles were visualized in a transmission electron microscope (TEM) (Table 3; Figure 1). The particles of *F. chica* + PVA had an average size of 245.5 nm, in the range of 200–300 nm for nanometric sizes. The PDI of the *F. chica* + PVA particles showed a value of 0.146, indicating monomodal and monodisperse particles, which tend to be more stable over time [39]. The blank nanoparticle generated a polydispersity index (PDi) of 0.7—values above 0.3 generally indicate widely polydisperse samples, which lead to instability in the system [40]

**TABLE 3:** Parameters of average size, PDi, and zeta potential of unloaded PVA film and *Fridericia chica* + PVA film evaluated by dynamic light scattering (DLS) assay.

	Unloaded PVA film	*F. chica* + PVA film
*φ* (nm)	475.3 ± 30.4	245.5 ± 0.9^*^
PDi	0.76 ± 0.06	0.146 ± 0.005^**^
*ζ* (mV)	−6.9 ± 0.25	−11.1 ± 0.2^***^

Results expressed as mean ± standard error of the mean (SEM) of 1 experiment in technical triplicate. Parameters: *φ* = average size; PDi = polydispersity index; *ζ* = zeta potential. *F. chica* + PVA film = PVA particles loaded with 2.5% of *F. chica* standard crude extract (SCE). Statistical analysis: paired *t*‐test. Unloaded PVA nanoparticles and *F. chica* + PVA parameters are significantly different (*p < 0.05; **p < 0.01; ***p < 0.001).

The zeta potential indicates the charge present on the surface of the particles and is a useful predictor of the system's stability, as well as the modification of surface properties during formulation preparation. For acceptable values of approximately |30 mV|, repulsive electrical forces predominate, which are sufficient to prevent particle aggregation. From |5| to |15| mV, limited flocculation occurs, with maximum flocculation between |5| and |3| mV [41, 42]. The blank PVA particle has a low negative value of −6.87 mV has relationship to the degree of polymerization and hydrolysis of the polymer [27, 41]. An −4.26 increase in negative charge after incorporation of *F. chica* extract decreased the zeta potential to −11.13 mV (Table 3). The presence of different compounds in the extract influenced the increase in negative charges [43].

One of the main problems with a low zeta potential is low electrostatic repulsion, which leads to increased aggregation of the nanoparticles [44]. Although the zeta potential is not close to the ideal value, there was no initial aggregation of the particles or increase in size. In fact, the particles were smaller after incorporating the crude extract. Future stability tests will be important to assess whether any changes occur over time. PVA, being a nonionic polymer, also influenced the low zeta potential value. Ghadiri et al. [45] obtained similar results when incorporating sweet almond oil nanoemulsion into PVA films, which showed a zeta potential of +13.5 mV. The presence of a nonionic surfactant in the formulation did not significantly contribute to an increase in this parameter. Improvements can be made by including components with appropriate charges to achieve higher potential. Servat‐Medina et al. [15] obtained a zeta potential of approximately +30 mV by associating the polymer chitosan with tripolyphosphate (TPP) to carry the *F. chica* crude extract. Despite the importance of zeta potential, system stability can also be achieved through steric repulsion, rather than electrostatic stabilization, which can lead to better overall stability [45].

Particle morphology was evaluated using TEM (Figure 1). The *F. chica* + PVA particles exhibited a spherical and regular shape, confirming a nanometric size of 200–300 nm. PVA can induce steric hindrance, reducing particle aggregation during the formulation process and producing smaller particles. The polymer may also interact with the extract's molecules, forming bonds that cover the particle surfaces, preventing contact between them and further reducing their average size [46].

### 2.4 Antifungal Activity

In the evaluation of antifungal activity, *F. chica* + PVA film effectively inhibited *Candida* species, showing greater inhibitory potential and lower MIC values than fluconazole (Table 4). In addition, *F. chica* + PVA film was able to inhibit the strains of *Staphylococcus aureus*, *Pseudomonas aeruginosa*, and *Escherichia coli* with MICs of 8.8 mg/mL and 16.7 mg/mL. Mafioleti et al. [24] evaluated the antimicrobial activity of the hydroethanolic extract against the same strains used in the present study. The extract produced by maceration in a 70% hydroethanolic solution for 7 days and the remaining solution, after solvent removal, dried by freeze drying did not inhibit growth at any of the concentrations tested. Torres et al. [47] obtained the extract by maceration in 80% ethanol for 7 days in the dark, at room temperature, and the results did not show the expected activity. However, Sousa et al. [32] when evaluating the antibacterial activity of *F. chica* SCE, obtained positive results only for *S. aureus*, with an MIC of 0.39 mg/mL. Therefore, *F. chica* + PVA film was able to enhance the antimicrobial effect of the extract, likely due to the improved bioavailability of the active compounds.

**TABLE 4:** In vitro minimum inhibitory concentration (MIC, mg/mL) for unloaded PVA film and *Fridericia chica* + PVA film.

Strains	MIC (mg/mL)			
	Unloaded PVA film	*F. chica* + PVA film	Positive controls	
			Fluconazole	Chlorhexidine
*Candida albicans*	*	0.059	0.25	n.a.
*Candida lusitanae*	*	0.059	0.5	n.a.
*Candida parapsilosis*	*	0.059	1.0	n.a.
*Candida rugosa*	*	0.030	0.25	n.a.
*Candida guilliermondii*	*	0.030	4.0	n.a.
*Candida glabrata*	*	0.015	8.0	n.a.
*Candida krusei*	*	0.030	16	n.a.
*Candida tropicalis*	*	0.059	0.5	n.a.
*Staphylococcus aureus*	*	8.3	n.a.	1.2
*Pseudomonas aeruginosa*	*	8.3	n.a.	1.2
*Escherichia coli*	*	16.7	n.a.	1.2

*Note*: Results of three independent assays, in eight technical replicates per concentration.

Abbreviations: MIC, minimum inhibitory concentration (lowest concentration (mg/mL) capable of inhibiting microbial growth); n.a., not applicable.

### 2.5 Cell Viability Assay

**FIGURE 2:**
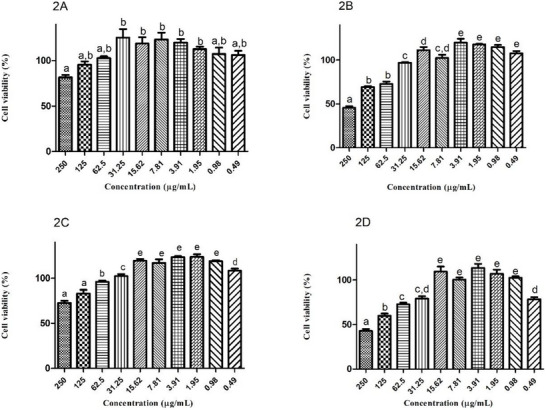
Influence of unloaded PVA film (A and C) and *Fridericia chica* + PVA film (B and D) on relative cell viability of HGF‐1 and HaCaT cell lines. Data were expressed as mean ± standard error of the mean (SEM) of two independent experiments with technical triplicate per concentration. Cell lines: HGF‐1 (human finite gingival fibroblasts; A and B); HaCaT (human immortalized keratinocytes; C and D). Samples: unloaded PVA film (A and C); *F. chica* + PVA film (B and D). Time exposure: 24 h (HaCaT); 48 h (HGF‐1). Sample concentration: 0.49–250 µg/mL. Statistical analysis: ANOVA and Tukey's test. Means followed by the same letter do not differ significantly by the Tukey's test at *p* < 0.05.

After 48 and 24 h of exposure, unloaded PVA film did not affect the viability of HGF‐1 and HaCaT cells up to a concentration of 250 µg/mL. However, *F. chica* + PVA film reduced cell viability to below 75% at concentrations above 62.5 µg/mL for both cell lines (Figure 2). Seba et al. [48] compared the cell viability of blank PVA films with doxorubicin and the compound 40‐amino‐1‐naphthyl‐chalcone in a colorimetric MTT assay (3‐(4,5‐dimethylthiazol‐2‐yl)‐2,5‐diphenyltetrazolium bromide) using osteosarcoma tumor cell lines. The blank particles did not reduce cell viability, demonstrating the low cytotoxicity of the PVA film at the tested concentrations. PVA is a water‐soluble polymer that is low‐cost, has low oral toxicity (LD_50_ = 15–20 g/kg; NOAEL = 5 g/kg/day), is biodegradable, and can form environmentally friendly membranes. The growing interest in the use of PVA in recent years is reflected in the increasing volume of publications in journals from 2000 to 2024 [49, 50].

Regarding *F. chica* SCE, Zago et al. [51] used HaCaT cell lines to evaluate cell viability using the exclusion dye sulforhodamine B after treatment with the crude extract. The extract maintained cell viability at 112.2% and 105.4% at concentrations of 5 and 10 µg/mL, respectively. The 50 µg/mL concentration was cytotoxic in this experiment. The decrease in cell viability after incorporating the extract into the PVA film is due to the intrinsic properties of the extract, without any influence from the film itself. The cytotoxicity of *F. chica* SCE at concentrations above 62.5 µg/mL may have relationship to the antioxidant potential of the plant, which, although desirable in the healing process, can also generate ROS during oxidation–reduction reactions. Moreover, studies have demonstrated the formation of hydrogen peroxide and other oxidation products from the interaction between phenolic compounds and components of the culture medium, causing cytotoxicity regardless of the cell line [52, 53]. Therefore, concentrations of *F. chica* + PVA film below 62.5 µg/mL were more suitable for cell viability maintenance, as they can sustain the balance between oxidizer and antioxidant agents.

In vivo models were analyzed to better evaluate the safety of the extract. In the study by Jorge et al. [14], the crude *F. chica* extract (100 mg/mL) was administered to wounds on Wistar rats with induced diabetes (200 µL/wound) for 10 consecutive days. The extract was able to reduce the wound area by 96% compared to the control group (36% healing). No adverse effects were reported at the administered dose. Servat‐Medina et al. [15] evaluated the healing potential of *F. chica* SCE nanoparticles in gastric ulcers at concentrations of 30, 100, and 300 mg/kg. The nanoparticle dose of 60 mg/kg of plant extract had the same gastroprotective effect as the highest dose of the crude extract (300 mg/kg). Moreover, the nanoparticles demonstrated the ability to maintain cell viability at lower concentrations and, at higher concentrations, promoted fibroblast proliferation, demonstrating that the nanoparticles could be useful to improve the extract's biocompatibility [15].

These same nanoparticles were incorporated into a 3% PVA film and applied to skin wounds on Wistar rats with induced diabetes. The lowest dose of the PVA film, loaded with 0.5 mg of *F. chica* nanoparticles, resulted in approximately 79% wound contraction. Throughout the experiment all animals gained weight, indicating that the lesion and treatment did not affect this parameter at any of the tested concentrations [54]. In a Phase I clinical study, the safety of the gel containing 2.5% *F. chica* SCE was evaluated. At the tested concentration, no adverse effects related to the treatment were reported [28]. This finding allowed the development of the treatment protocol, which was subsequently applied in a Phase II/III clinical trial [4].

### 2.6 Ex Vivo Permeation Assay

**FIGURE 3:**
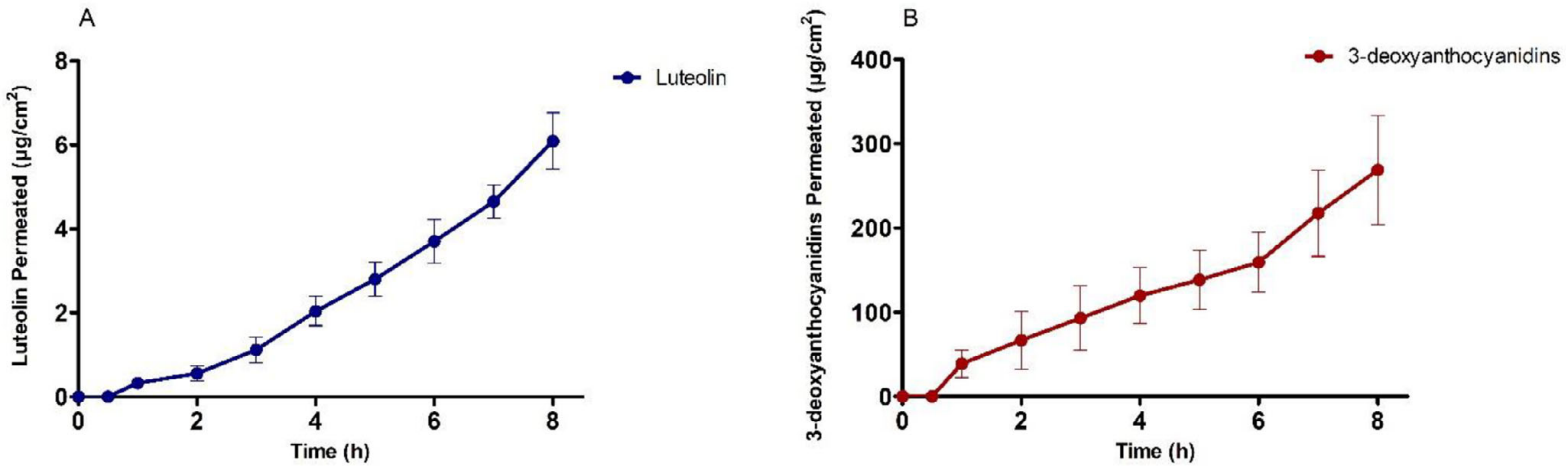
Permeation profile of luteolin (A) and 3‐deoxyanthocyanidins (B) in porcine esophageal mucosa. Data expressed as mean ± standard error of the mean (SEM) of six authentic replicates for each permeation time. Permeation profile expressed in µg/cm^2^ versus time (h).

According to data (Figure 3), both compounds permeated efficiently through porcine esophageal mucosa, demonstrating a continuous supply of active ingredients to the mucosa with constant flow. The permeability coefficient (*P*) and flux (*J*) were higher for 3‐deoxyanthocyanidins (15.2 ± 0.86.10^3^cm^2^/h and 30.3 ± 1.71 µg/cm^2^h) when compared to luteolin (1.3 ± 0.04.10^3^cm^2^/h and 0.81 ± 0.03 µg/cm^2^h) (Table 5). The amount of luteolin recovered and retained in the mucosa was 226.51 ± 23.90 and 13.05 ± 1.21 µg, respectively. The values of 3‐deoxyanthocyanidins recovered and retained were higher: 540.96 ± 28.65 and 37.56 ± 3.61 µg, respectively (Table 5).

**TABLE 5:** Permeation parameters of luteolin and 3‐deoxyanthocyanidins after application of *Fridericia chica* + PVA film on porcine esophageal mucosa.

Parameters	Compounds	
	Luteolin	3‐Deoxyanthocyanidins
*J* (µg/cm^2^h)	0.81 ± 0.03	30.3 ± 1.71^***^
Time lag (h)	1.0 ± 0.12	0.3 ± 0.05^**^
*P* (10^3^cm^2^/h)	1.3 ± 0.04	15.2 ± 0.86^***^
*R* ^2^	0.9696	0.9666
Amount recovered (µg)	226.51 ± 23.90	540.96 ± 28,65^***^
amount retained (µg)	13.05 ± 1.21	37.56 ± 3.61^***^

*Note*: Results expressed as mean ± standard error of the mean (SEM) of six replicates in nine different times (from 0.5 min to 8 h). Parameters: *J* = penetration flux; *P* = permeability coefficient; *R* ^2^ = coefficient of determination (linear regression model). Statistical analysis: paired *t*‐test. The values in the columns for each parameter are significantly different (**p < 0.01; ***p < 0.001).

When this conventional film is combined with nanoparticles for wound treatment, new therapeutic options may emerge, offering benefits such as increased versatility and controlled release of therapeutic agents [55]. An advantage of the mucoadhesive film over the gel used in the clinical study is its improved adhesion, leading to a larger contact area with the mucosa and, consequently, a reduction in the number of times required for product administration. The PVA polymer also contains a significant number of hydroxyl groups along the polymer's main chains, promoting hydrogen bonding. As a result, the film can facilitate the permeation of the extract into the environment. However, films with semi‐synthetic and synthetic bioadhesive polymers can delay the elimination of drugs by the body [56]. In the case of *F. chica* + PVA film (Table 5; Figure 3), the mechanical properties of the film were positive and contributed to the sample's continuous release.

## 3 Conclusions

The *F. chica* + PVA film demonstrated antioxidant activity, in addition to antimicrobial potential against *Candida* spp. and bacteria strains. This pharmacological potential is associated with the phenolic composition of the extract, particularly the presence of 3‐deoxyanthocyanidins. The film did not affect the cell viability of human gingival fibroblasts (HGF‐1) and immortalized human keratinocytes (HaCaT) up to the concentration of 62.5 µg/mL, with concentration‐dependent cytotoxicity.

The film containing the extract showed nanometric size particles, monodisperse characteristics, and reduced zeta potential. Luteolin and 3‐deoxyanthocyanidins permeated porcine esophageal membranes easily and continuously, demonstrating that PVA facilitated delivery without negatively affecting compound release. This association can be useful for the treatment of mucositis as the film adhered efficiently to the affected mucosa, promoting faster healing.

The convenient application of biofilms ensures greater accessibility and autonomy to patients affected by mucositis, preventing the aggravation of injuries and health conditions. Future studies will be required to assess the long‐term stability of *F. chica* + PVA film, ensuring practical application and appropriate shelf‐life outcome. Moreover, clinical validation will be crucial for evaluating the product's effectiveness and safety, addressing the limitations of the current study and confirming the product's applicability in the treatment of mucositis.

## 4 Experimental Section

### 4.1 Reagents

2,2‐Diphenyl‐1‐picrylhydrazyl (DPPH), Folin–Ciocalteu, gallic acid, catechin, quercetin, Trolox and dimethyl sulfoxide (DMSO) were purchased from Sigma‐Aldrich (St. Louis, MO, USA). PVA was from Dinâmica. HPLC‐grade ethanol was Merck and ultrapure water produced by the Milli Q system (Millipore Corporation, Billerica, MA, USA).

### 4.2 Plant Material and Extract Preparation

*F. chica* leaves (accession 06, voucher specimen 1865) were collected from the Germplasm Bank at the experimental field of the Multidisciplinary Center for Chemical, Biological, and Agricultural Research (CPQBA/UNICAMP), Paulínia, São Paulo, Brazil, in the morning during March 2014. The voucher was identified by Professor Maria do Carmo Estanislau do Amaral from the Botany department at Biology Institute at Unicamp. Authorization for access to genetic heritage was registered in the National System for the Management of Genetic Heritage and Associated Traditional Knowledge (SisGen) under number 010150/2012‐9.

*F. chica* hydroalcoholic standardized extract was produced according to the method described by Servat‐Medina et al. [15] and Sousa et al. [32]. The leaves were dried for 48 h in an oven at 40°C (211 model—Fabbe), with forced ventilation and then ground in a hammer mill (UM 40 model—Stephen) using a 40‐mesh sieve. One kilogram of the dried material was transferred to a 50 L stainless steel tank with mechanical stirring, using a 70% ethanol + 0.3% citric acid extraction solvent in a 1:5 (w/v) ratio (plant:solvent). This process was repeated three times, with each cycle lasting approximately 90 min. The resulting extract was concentrated under vacuum using a rotary evaporator (R‐200 model—Büchi) until the final volume was reduced by 80%. The resulting crude extract obtained was dried in a spray dryer (Model B‐290, loop B‐295—Büchi) with the following settings: inlet temperature: 100°C ± 2°C; outlet temperature: 60°C ± 2°C; flux: 250 mL/h; aspiration flow: with N_2_; injection pressure: 414 L/h; and feed flow: 5 mL/min at room temperature. *F. chica* hydroalcoholic crude standardized extract provided a 10% extraction yield and was stored under refrigeration in aluminum packaging until analysis [32].

### 4.3 Preparation of Films

**FIGURE 4:**
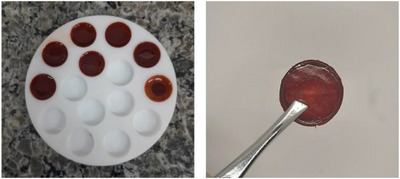
Image of PVA film containing *F. chica* SCE.

A 3% PVA (MW = 44 053) solution was stirred and heated to 90°C for 48 h, then filtered under vacuum using a Büchner funnel with porosity No. 4. The incorporation of crude extract (2.5% w/v) into the PVA solution was performed using a tip sonicator (Q500 model—QSonica) with a 17 mm cylindrical titanium alloy probe. The program was set with a pulse duration of 30‐s on/30‐s off and an amplitude of 55% for 2 min. This process was repeated three times, totaling 6 min of preparation. One milliliter (1 mL) of the resulting solution was deposited into polytetrafluoroethylene (PTFE) molds and heated in an oven at 40°C for solvent evaporation, resulting in the formation of circular films with a diameter of 1 cm, each containing 25 mg of *F. chica* SCE. The image of the film containing *F. chica* SCE can be seen in Figure 4 below.

### 4.4 PVA Film Characterization

#### 4.4.1 Particle Size, PDi, and Zeta Potential

The mean particle diameter and PDi were determined using the DLS technique with Zetasizer analyzer (Zetasizer nano ZS ZEN 3600—Malvern). Aliquots of the *F. chica* + PVA and unloaded PVA suspensions, reserved before oven drying, were previously diluted (1:100) in purified water and individually placed in capillary cells (DTS1070—Malvern). All determinations were performed in triplicate.

#### 4.4.2 TEM

The morphology of the *F. chica* + PVA film particles was analyzed at the Imaging and Microscopy Center of the Piracicaba Dental School (FOP/UNICAMP) using a transmission electron microscope (JEM‐1400 model—JEOL) operating at 200 kV. A drop of the sample suspensions, obtained from aliquots reserved before the oven‐drying process, was previously deposited onto formvar/carbon‐supported copper grids (300 mesh) and dried at room temperature before visualization.

### 4.5 Chemical Characterization

#### 4.5.1 Total Phenolic and Flavonoid Content

The total phenolic and flavonoid content was estimated according to procedure described by Costa et al. [57] with modifications. A serial dilution of 2.7 mg *F. chica* + PVA film in the range of 1000–31.25 µg/mL was prepared, and 30 µL aliquots of each dilution were transferred to 96‐well plates. The same procedure was performed for *F. chica* SCE. Standard calibration curves for the phenolic compounds and flavonoids were constructed using gallic acid (linearity = 5–100 µg/mL; *R* ^2^ = 0.9911 ± 0.0115) and catechin (linearity = 5−300 µg/mL; *i* ^2^ = 0.9965 ± 0.0021) with absorbance at 765 and 510 nm, respectively, using a microplate reader (Synergy HT—B‐SHT, Biotek). Three independent trials were conducted, with triplicates at each concentration. The results are expressed as milligrams of gallic acid equivalent per gram of sample (mg GAE/g sample) and per milligram of catechin equivalent per gram of sample (mg CAE/g sample), respectively.

### 4.6 In Vitro Biological Activities

#### 4.6.1 Scavenging of DPPH Free Radicals

Serial dilutions (1000–31.25 µg/mL) of 2.7 mg of *F. chica* + PVA film and *F. chica* SCE were performed [57]. The reduction of the DPPH radical was evaluated by measuring absorbance at 517 nm. Antioxidant activity was expressed as the percentage of DPPH discoloration, calculated using equation below.

$$\%=\left[\left(\left(A_{\mathrm{DPPH}}-A_{S}\right)/A_{\mathrm{DPPH}}\right)\right]\times 100$$

In which *A*_S_ is the absorbance of the solution containing the extract and *A*_DPPH_ is the absorption of the DPPH solution. Three independent trials were conducted, with triplicates at each concentration. The extract concentration required to achieve 50% antioxidant activity (IC_50_) was determined from the plot of antioxidant percentage versus extract concentration, using Microsoft Excel software.

#### 4.6.2 Antioxidant Potential Assay (FRAP)

The ferric reducing antioxidant potential (FRAP) was determined using the method described by Che et al. [58] with modifications. Experiments with 2.7 mg of *F. chica* incorporated in PVA film and with *F. chica* SCE were carried out at a sample concentration of 125 µg/mL. The reaction mixture incubated at 37°C for 30 min had absorbance read at 595 nm. The calibration curve was constructed with known concentrations of 1 mM ferrous sulfate (linearity range: 25–500 µM, *R* ^2^ > 0.998) diluted with distilled water and used to correlate the sample absorbance and the concentration of the standard solution. The results, with three independent assays performed in triplicate for each analysis, are expressed in µM equivalent of ferrous sulfate per gram of sample (µM FSE/g).

#### 4.6.3 Scavenging Capacity of ROS

The *F. chica* SCE, *F. chica* + PVA film, and the quercetin and gallic acid standards, previously dissolved in phosphate buffer and diluted to different concentrations (1000–31.25 µg/mL), were tested for ROS. Scavenging assays of superoxide anion radical (O_2_^−^), hydrogen peroxide (H_2_O_2_), and hypochlorous acid (HClO) were performed by spectrophotometry (Synergy HT—B‐SHT, Biotek) equipped with a thermostat for fluorescence, UV/Vis, and chemiluminescence measurements. Three independent trials were conducted that provided the inhibition percentage curve versus antioxidant concentration curve [59]. Details of this experiment can be found in Supporting Information (Document S4).

#### 4.6.4 Antimicrobial Evaluation

The unloaded PVA film and *F. chica* + PVA film were assessed for antifungal and antibacterial activity at the Microbiology Laboratory of Piracicaba Dental School, State University of Campinas (FOP‐UNICAMP). The strains used included *Candida krusei* (CBS 573), *Candida tropicalis* (CBS 94), *Candida guilliermondii* (CBS 566), *Candida parapsilosis* (CBS 604), *Candida albicans* (ATCC 90028), *Candida glabrata* (ATCC 5207), *Candida lusitaniae* (IZ 06), *Candida rugosa* (IZ 12), *E. coli* (ATCC 35218), *S. aureus* (ATCC 29213), and *P. aeruginosa* (ATCC 27853).

The samples' minimum inhibitory concentrations (MICs) were determined using the broth microdilution method with Müeller–Hinton broth (MHB) for bacteria and RPMI‐1640 medium for fungi [60, 61]. Three independent experiments were conducted for each assay. Details of this experiment can be found in Supporting Information (Document S1).

#### 4.6.5 Cell Viability Assay

The immortalized human keratinocyte cell line HaCaT (CLS—Cell Lines Service, number 300493) was kindly provided by Piracicaba Dental School (FOP/UNICAMP), and human gingival fibroblasts (HGF‐1 ATCC CRL‐2014) were generously donated by Prof. Dr. Ramiro M. Murata from East Carolina University (ECU), Greenville, NC, USA.

Two independent analyses were performed for each sample, with triplicates for each concentration. The experiment was carried out according to Braga et al. [62]. The complete experimental protocol is available in Supporting Information (Document S2).

### 4.7 Franz Vertical Diffusion Cell Permeation Assay

A certified abattoir by the Department of Agriculture and Food Supply of São Paulo (Angelelli Ltda, Piracicaba, SP) provided porcine esophageal membranes (*Sus scrofa domesticus*, Landrace breed, 5‐month‐old, weighing approximately 75–80 kg). The experiments were conducted using a Franz vertical diffusion cell [63, 64] (Hanson Research Corporation, Chatsworth, CA, USA). In accordance with the Brazilian Guidelines for the Care and Use of Animals in Teaching or Scientific Research Activities (DBCA), Normative Resolution Num. 55, submission of an experimental protocol to the Brazilian Institutional Animal Care and Use Committee (CEUA) is not required for biological sample leftovers obtained from services such as abattoirs (CEUA exemption certificate No. 114/2025).

The solvent systems were according to the options proposed in the protocol described by the Organization for Economic Cooperation and Development (OECD) [65]. Six authentic replicates for each permeation time were performed. The complete permeation experimental protocol can be found at Supporting Information (Document S3).

### 4.8 Statistical Analysis

Data are expressed as the mean ± standard error of the mean (SEM). Statistical analysis was performed using paired Student's *t*‐test. A significance level of *p* < 0.05 was considered to assess differences between groups. Graph were generated using GraphPad Prism 5.0 (GraphPad Software, Inc., San Diego, CA, USA).

## Author Contributions

**Ilza Maria de Oliveira Sousa**: original draft, investigation, formal analysis, data curation. **Ailane Souza de Freitas**: writing, review, editing, data curation. **Fabrício de Faveri Favero**: writing, review, editing, data curation. **Diana Pinto**: investigation, data curation. **Mariana Cecchetto Figueiredo**: investigation, data curation. **Lucia Elaine O. Braga**: investigation, data curation. **Simone Nataly Busato de Feira**: investigation, data curation. **Nubia de Cássia Almeida Queiroz**: data curation. **Ana Lucia T. G. Ruiz**: design of methodology, formal analysis, data curation. **José Francisco Hoflin**: data curation. **João Ernesto de Carvalho**: formal analysis, data curation. **Mary Ann Foglio**: funding acquisition, supervision, investigation, conceptualization.

## Funding

This work is sponsored by CNPq (grant number: 301724/2022‐9), CAPES, FINEP, and FAPESP (Grant number: 2021/01280‐3).

## Conflicts of Interest

The authors declare no conflicts of interest.

## Supporting information




**Supporting File 1**: cbdv70619‐sup‐0001‐SuppMat.docx

## Data Availability

The data that support the findings of this study are available in the supplementary material of this article.
