# Supplementary material for: Mucoadhesive Polyvinyl Alcohol Film Containing Fridericia chica Extract for Mucositis Treatment: Pharmacological Evaluation and Active Compounds Release
Source: Chem Biodivers. 2025 Oct 25;23(1):e02095. doi: 10.1002/cbdv.202502095 (PMC12761349; doi:10.1002/cbdv.202502095)
Supplement: Supplementary file 1 — Supporting File 1: cbdv70619‐sup‐0001‐SuppMat.docx [file CBDV-23-e02095-s001.docx]

**Chemistry and Biodiversity**

**Supporting Information**

**Mucoadhesive Polyvinyl Alcohol Film Containing *Fridericia chica* Extract for Mucositis Treatment: Pharmacological Evaluation and Active Compounds Release**

Ilza Maria de Oliveira Sousa^1^, Ailane Souza de Freitas^2^, Fabrício de Faveri Favero^2,3^, Lucia Elaine O. Braga^2^, Mariana Cecchetto Figueiredo^1^, Diana Pinto^4^, Maria Beatriz P.P. Oliveira^4^, Simone Nataly Busato de Feiria^3^, Nubia de Cássia Almeida Queiroz^1^, Ana Lucia T.G. Ruiz^2^, José Francisco Hofling^3^, João Ernesto de Carvalho^2^, Mary Ann Foglio^2*^.

**Mucoadhesive Polyvinyl Alcohol Film Containing *Fridericia chica* Extract for Mucositis Treatment: Pharmacological Evaluation and Active Compounds Release**

Ilza Maria de Oliveira Sousa^1^, Ailane Souza de Freitas^2^, Fabrício de Faveri Favero^2,3^, Lucia Elaine O. Braga^2^, Mariana Cecchetto Figueiredo^1^, Diana Pinto^4^, Maria Beatriz P.P. Oliveira^4^, Simone Nataly Busato de Feiria^3^, Nubia de Cássia Almeida Queiroz^1^, Ana Lucia T.G. Ruiz^2^, José Francisco Hofling^3^, João Ernesto de Carvalho^2^, Mary Ann Foglio^2*^.

^1^Graduate Program in Medical Sciences – School of Medical Science (FCM), Universidade Estadual de Campinas (UNICAMP), Campinas, SP, Brazil

^2^School of Pharmaceutical Sciences (FCF), Universidade Estadual de Campinas (UNICAMP), Campinas, SP, Brazil, *e-mail: foglioma@unicamp.br

^3^Department of Physiological Science – Piracicaba Dental School (FOP), Universidade Estadual de Campinas (UNICAMP), Piracicaba, SP, Brazil

^4^LAQV/REQUIMTE, Faculty of Pharmacy, University of Porto, Porto, Portugal

**Table of contents**

**Document S1 – Proceeding of antimicrobial evaluation of the PVA films3**

**Document S2 – Experimental protocol of cell viability 4**

**Document S3 – Experimental protocol of Franz vertical diffusion cell permeation 5**

**Document S4 – Scavenging capacity of reactive species (ROS)7**

**Document S1 – Proceeding of antimicrobial evaluation of the PVA films**

The antifungal and antibacterial activities of the films were tested against different *Candida* strains (*Candida krusei* CBS 573, *Candida tropicalis* CBS 94, *Candida guilliermondii* CBS 56, *Candida parapsilosis* CBS 604, *Candida albicans* ATCC 90028, *Candida glabrata* ATCC 5207, *Candida lusitaniae* IZ 06, *Candida rugosa* IZ 12) and bacteria (*Escherichia coli* ATCC 35218, *Staphylococcus aureus* ATCC 2921, and *Pseudomonas aeruginosa* ATCC 27853), using the broth microdilution method, according to the Clinical and Laboratory Standards Institute protocols M26-A and M27 [60,61].

For antifungal activity evaluation, the inoculum of each strain was diluted in saline solution (0.9% NaCl), and the turbidity was adjusted to 0.5 on the McFarland scale using spectrophotometric readings (Synergy HT – B-SHT, Biotek®, absorbance between 0.08 and 0.10) at 530 nm, corresponding to a final cell concentration of 2.5 × 10⁴ CFU/mL.

RPMI-1640 medium was dispensed into sterile 96-well plates (100 µL/well), with three plates prepared for each sample. Solutions of unloaded PVA film and *F. chica* + PVA film (0.56 mg/mL, 100 µL/well) were added, followed by serial dilution at a 1:2 ratio, resulting in a total of 12 concentrations (eight replicates for each concentration). *Candida* spp. inocula (100 µL/well) were then added.

For evaluation of the samples against the selected bacteria, inocula were prepared in saline solution, adjusting the turbidity to 0.5 on the McFarland scale, corresponding to an absorbance of 0.08 to 0.10 at 625 nm on the spectrophotometer (Synergy HT – B-SHT, Biotek®,). The final cell concentration in the plate was 5.0 × 10⁵ CFU/mL.

The initial concentrations of the samples were 33.3 mg/mL for *F. chica* + PVA film and 30 mg/mL for unloaded PVA film. In a sterile 96-well microplate, 100 µL of Müller-Hinton medium and 100 µL of the sample solutions were added, followed by serial dilutions. Subsequently, 100 µL of the previously prepared inoculum was added to each well. Three independent experiments were conducted for each sample.

Chlorhexidine at a concentration of 0.12% was used as a positive control for bacteria, and fluconazole (0.25–8 mg/mL) as a positive control for *Candida* spp. Culture media supplemented with dimethyl sulfoxide (DMSO < 1%) were used as negative controls. All plates were incubated for 24 h (bacteria) or 48 h (fungi) at 37°C in aerobic conditions. At the end of the incubation period, 30 µL of resazurin solution was added to each well. After 2 h, visual observation of color development (pink color, indicative of viable microorganisms) was performed, and the Minimum Inhibitory Concentration (MIC) was defined as the lowest concentration of the sample capable of inhibiting visible microorganism growth (the lowest concentration showing a blue color).

**Document S2 – Experimental protocol of cell viability**

Human keratinocyte cell lines (HaCaT CLS – Cell Lines Service, catalog number 300493) and human gingival fibroblasts (HGF-1 ATCC® CRL-2014™) were cultured in 25 cm² cell culture flasks containing 5 mL of RPMI 1640 medium (Gibco®), supplemented with 5% fetal bovine serum (FBS, Gibco®) and 0.1% penicillin:streptomycin (1:1). The cells were maintained at 37°C in a humidified atmosphere with 5% CO_2_. Experiments were conducted between passages 5 and 10 after thawing.

The experiment was carried out according to Braga et al.[62]. Cell densities were adjusted to 4 × 10⁴ and 1 × 10⁵ cells/mL, respectively. A volume of 100 µL of each cell suspension was applied to 96-well plates, which were incubated for 24 h at 37°C in a humidified atmosphere with 5% CO_2_. Aliquots of unloaded PVA film and *F. chica* + PVA film particles were dissolved in a DMSO solution (Merck®) at a concentration of 100 mg/mL, corresponding to the extract, and then serially diluted in complete medium to achieve final concentrations ranging from 0.49 to 250 µg/mL. Samples (100 µL/compartment) were applied in triplicate, and cells were incubated at 37°C in a 5% CO_2_ atmosphere with a humidified environment. Based on the doubling time of each cell line, incubation times were 24 h for HaCaT cells and 48 h for HGF-1 cells. Following exposure, cells were fixed with 50% trichloroacetic acid (Sigma®) and stained with 0.4% Sulforhodamine B (Sigma®) in acetic acid. Spectrophotometric measurements were taken at 540 nm using a microplate reader (Molecular Devices®, VersaMax model). Relative cell viability (%) was calculated for each concentration tested, considering untreated cells (blank sample) as representing 100% viability, using Equation 1:

Cell viability (%) = 100 x [(ODS – ODB) / (ODC)] (Eq. 1)

ODS = Optical density of the sample; ODB = Optical density of the blank sample; ODC = Optical density of control (untreated cells)

**Document S3 – Experimental protocol of Franz vertical diffusion cell permeation**

Fresh porcine esophagi (*Sus scrofa domesticus*) donated by certified butchers were transported to the pharmacology laboratory at CPQBA in phosphate-buffered saline (PBS) solution within 2 h of collection. A scalpel was used to carefully separate the esophageal mucosa from the surrounding tissue, discarding any visibly damaged areas of the mucosal surface. The epithelium was immediately immersed in deionized water at 60°C for 2 min after separation from the connective tissue. The epithelium was then placed on a 15 cm cellulose filter, with the connective side facing the membrane filter to prevent damage, given the tissue's fragility. The solvent systems used were based on the options outlined in the protocol described by the Organization for Economic Co-operation and Development (OECD) [65].

The experiments were conducted using Franz vertical diffusion cells (Hanson Research Corporation, Chatsworth, CA, USA). The porcine mucous membranes were washed and equilibrated with phosphate buffer at pH 7.0 for approximately 10 min before being assembled between the donor and receptor compartments of the static vertical Franz diffusion cells (receptor phase volume: 4.5 mL; diffusion area: 0.60 cm²). Sample collection times were 0 and 30 min, and 1, 2, 3, 4, 5, 6, 7, and 8 h, resulting in a total of 10 collections for each sample. An ethanol:water solution (20:80) was used as the receptor phase, constantly stirred with a small magnetic bar at 600 rpm and maintained at 36±1°C. The samples (circular films with a diameter of 1 cm, each containing 25 mg of crude extract) were evenly applied to the membrane surface in the donor compartment and sealed with a Parafilm® membrane to prevent evaporation.

The content of 3-deoxyanthocyanidins present in the formulation, permeated and retained in the mucosa, was measured by spectrophotometry in the ultraviolet region (Synergy HT – B-SHT, Biotek®), with absorbance measurements at 470 nm. Luteolin was quantified using HPLC (Agilent^®^ 1100 series) equipped with a UV-VIS detector, automatic injector, and quaternary pumps, employing a C-18 reverse-phase column (250 mm x 4.6 mm x 5 µm, Phenomenex® Gemini), with a gradient mobile phase of methanol/H2O acidified with 0.1% formic acid. The mobile phase gradient programming (A/B) was as follows: 0-5 min, 55%/45%; 5-28 min, 30%/70%; 28-35 min, 10%/90%; and 35-45 min, 55%/45%. Injection volume: 20 µL; mobile phase flow rate: 1 mL/min; detection wavelength: 345 nm. The trials were conducted with 6 authentic replicates for each permeation time evaluated.

The samples were collected immediately after application (time 0) and at pre-determined time intervals, with the respective volume replaced by an equivalent amount (300 µL) of receptor medium. The permeation flux (J) was obtained by the slope coefficient of the line that relates the amount permeated to time (µg /cm²·h). The lag time (h), defined as the time required for the substance to reach equilibrium when passing through the membrane, was calculated by extrapolating the stationary phase line.

The permeability coefficient (P, expressed in 10^3^cm²/h) was determined by dividing the permeation flux (J, expressed in µg/cm².h) by the initial drug concentration in the donor compartment (expressed in µg/cm³), applying Fick's second law of diffusion. It was assumed that under sink conditions, the drug concentration in the receptor compartment is negligible compared to that in the donor compartment.

The non-permeated material corresponds to the sample retained in the esophageal mucosa, and the recovered material remained in the stratum corneum. To assess non-permeated material, the esophageal mucosa exposed to the formulation for 8 h, under the conditions previously described, was used. At the end of the experiment, excess sample was removed with absorbent paper and supported on a watch glass to avoid losses. The recovered material was then reserved for compound quantification. For the evaluation of retained material, the mucosa was scored using scissors, and the fragments were transferred to a tube for centrifugation, adding 5 mL of extracting solution (ethanol). The tubes were vortexed for two min, then processed in an Ultra Turrax homogenizer (T10 model - IKA®) for 1 min and finally treated with ultrasound for 30 min. Aliquots of 5 mL were also collected for the quantification of retained material.

The kinetic model evaluation was performed through mathematical treatment of the results obtained and determination of the reaction order: zero order (concentration versus time), first order (log of concentration versus time), and the Higuchi model (concentration versus the square root of time).

**Document S4 – Scavenging capacity of reactive species (ROS)**

The *F. chica* SCE, *F. chica* + PVA film, and the quercetin and gallic acid standards, previously dissolved in phosphate buffer and diluted to different concentrations (1000 µg/mL to 31.25 µg/mL), were tested for reactive species. Scavenging assays of superoxide anion radical (O_2_^−^), hydrogen peroxide (H_2_O_2_), and hypochlorous acid (HClO) were performed by spectrophotometry (Synergy HT – B-SHT, Biotek®) equipped with a thermostat for fluorescence, UV/Vis, and chemiluminescence measurements. Three independent trials were conducted that provided the inhibition percentage curve versus antioxidant concentration curve.

**Superoxide Anion (O₂•⁻) Scavenging Assay**

The ability of *F. chica* SCE and *F. chica* + PVA film to neutralize superoxide radicals was evaluated using the protocol described by Sousa et al^24^. The superoxide radicals were generated via the NADH/PMS system in a final reaction volume of 300 μL, composed of NADH (166 μM, 50 μL), NBT (43 μM, 150 μL), 50 μL of *F. chica* SCE or *F. chica* + PVA film (ranging from 1000 µg/mL to 31.25 µg/mL), and PMS (2.7 μM, 50 μL), all diluted in 19 mM potassium phosphate buffer (pH 7.4). The assay was conducted at 25 °C, and absorbance at 560 nm was recorded over 6 min (Synergy HT – B-SHT, Biotek®). The negative control consisted of buffer in place of the sample, and the blank was prepared by replacing both PMS and sample with buffer. Results were expressed as IC₅₀, indicating the concentration (μg/mL) required to inhibit 50% of superoxide radicals.

**Hydrogen Peroxide (H₂O₂) Scavenging Assay**

The H₂O₂ scavenging potential was determined based on the inhibition of chemiluminescence generated by the ROO•- induced oxidation of lucigenin, according to the method reported by Sousa et al^24^. Luminescence was monitored at 1-min intervals for a total duration of 5 min at 37 °C. The negative control included 19 mM phosphate buffer (pH 7.4), 50 mM Tris-HCl buffer (pH 7.4), lucigenin (800 μM in Tris buffer), and hydrogen peroxide (30% diluted in phosphate buffer).

**Hypochlorous Acid (HOCl) Scavenging Assay**

The HOCl scavenging activity was assessed by measuring the inhibition of dihydrorhodamine (DHR) oxidation to rhodamine 123, as induced by HOCl, following the procedure outlined by Sousa et al^24^. HOCl was freshly prepared from a 1% NaOCl solution, with the pH adjusted to 6.2 using 10% H₂SO₄. The reaction mixture (final volume of 300 μL) consisted of 50 μL of either *F. chica* SCE or *F. chica* + PVA film at varying concentrations, 150 μL of 100 mM phosphate buffer (pH 7.4), 50 μL of 5 μM DHR, and 50 μL of 30 μM HOCl. The assay was carried out at 37 °C, and fluorescence was immediately measured (Synergy HT – B-SHT, Biotek®, excitation at 485 nm; emission at 528 nm). Results were reported as IC₅₀ values (μg/mL), representing the concentration of extract required to inhibit 50% of HOCl-induced oxidation.
